# Joint estimation of point spread function and molecule positions in SMLM informed from multiple planes

**DOI:** 10.1364/BOE.551278

**Published:** 2025-03-04

**Authors:** Julian G. Maloberti, Lukas Velas, Simon Moser, Anna Gaugutz, Marina Bishara, Gerhard J. Schütz, Alexander Jesacher

**Affiliations:** 1 Institute of Biomedical Physics, Medical University of Innsbruck, Müllerstraße 44, 6020 Innsbruck, Austria; 2 Institute of Applied Physics, TU Wien, Getreidemarkt 9, 1060 Vienna, Austria

## Abstract

The advent of single molecule localization microscopy (SMLM) has transformed our capacity to investigate biological structures at the nanoscale. While the research focus has long been on improving localization precision, systematic errors caused by optical aberrations are often overlooked. In the case of 3D SMLM, such errors have the potential to significantly impair the quality of the resulting images. In this paper, we present an imaging and data processing approach that jointly estimates both, molecule positions and optical aberrations in SMLM. Therefore, the method minimizes systematic errors in SMLM reconstructions without the necessity of additional experimental calibration steps, such as the recording of fluorescent bead z-stacks. We investigate the reliability of this approach, especially in situations where the joint retrieval can be expected to be ill-posed, i.e., whenever the sample is "flat" and provides little diversity among the captured single molecule images. To enhance the reliability of the inverse problem solution, we suggest utilizing small SMLM data sets acquired at one or more slightly defocused "auxiliary" planes. We investigate the effectiveness of our approach through numerical simulations and imaging experiments of a calibration probe and nuclear pore complexes. Our method is simple and integrates seamlessly into existing SMLM setups without necessitating modifications or added complexity to the system.

## 1. Introduction

Single molecule localization microscopy (SMLM) is a powerful optical microscopy concept that enables spatial resolutions in the nanometer range [1]. It is predominantly used for fluorescent probes and is based on taking a series of wide-field images of the specimen, each containing only a sparse random subset of the entity of dye molecules. The positions of all molecules are inferred by fitting a molecule image model to the experimental recordings and the map of all positions represents a super-resolved image of the specimen.

Molecule localization accuracy and precision are important quality criteria of SMLM recordings. Precision affects the spatial resolution of the final SMLM image. It is defined as the positional standard error of a group of photons emitted by a single molecule. The precision depends on the shape of the microscope’s point spread function (PSF), which should be as compact as possible, as well as the signal to noise and signal to background ratios. Accuracy, on the other hand, describes systematic errors (biases) which may lead to image distortions and usually arise from an inadequate model of the PSF. Utilizing a highly accurate PSF model is particularly important for 3D localizations in SMLM.

Although modern microscopes have an excellent imaging performance, there still exist differences between theoretically calculated and experimental PSFs that are significant with regard to the high accuracy one seeks to obtain in SMLM. These differences can arise from the optical setup, the specimen or both. Examples for the former category are field dependent aberrations or spherical aberrations, e.g. introduced by temperature variations. Examples for the latter are refractive index (RI) inhomogeneities of the sample which distort the PSF, or wrong assumptions on the orientation of the fluorophore’s emission dipoles.

A common strategy to minimize biases in SMLM is to carefully characterize the PSF in a separate calibration step, e.g. by recording images of small fluorescent beads at different defocus values, followed by fitting a 3D image model to this stack with a parameterized phase pattern in the pupil plane [2–9]. Alternative approaches use spline models of PSFs that are directly recorded from bead z-stacks [10]. More recent works explored the use of neural networks [11] and expanded the phase retrieval concept to the recovery of polarization dependent pupil functions [12].

However, despite their usefulness, calibration approaches that utilize beads have a certain shortcoming: While they can well address effects of the optical system, they are unable to provide information about unknown sample aberrations, as for this they must be spread over the entire sample volume. Furthermore, bead calibrations add additional manual steps to the imaging process, such as adding beads to the sample during the preparation step.

Recent research has addressed the possibility of retrieving the PSF shape jointly with the molecule position data [7,13–17]. Such methods have become feasible thanks to highly parallelized GPU computing. However, despite being powerful and convenient for the reasons mentioned above, they also have a pitfall: as they are essentially blind deconvolution strategies, they are at risk to fail if the available data are rendering the problem not sufficiently well posed. In the specific context of SMLM, we hypothesize that blind deconvolution is particularly prone to failure when the sample has insufficient extent along the optical axis (z-axis). In such cases, the collected molecule images may contain insufficient information about the PSF and the algorithm may produce wrong estimates. Unfortunately, such “flat” samples are regularly targeted with SMLM, for example structures attached to cellular membranes or any objects imaged in a TIRF (Total Internal Reflection Fluorescence) setting.

In this paper, we investigate if and under which imaging conditions approaches of joint position and aberration estimation are at risk to fail. In particular, we investigate the roles of a limited axial sample extent, the signal to background ratio (SBR) and the cumulative signal, i.e. the total number of photons collected. We find our hypothesis confirmed that flat samples are more challenging. For such ill-posed situations we propose a strategy to increase the robustness: In analogy to the recording of multiple bead images at different defocus values when estimating aberrations from a bead z-stack, we propose to record short SMLM image series at defocused image conditions in addition to the main recording and to process the entire data in a dedicated gradient descent algorithm that produces estimates for molecule positions and aberrations. We note that the strategy can be viewed as a realization of the “phase diversity” concept which has been developed in the context of astronomical imaging [18,19]. We can confirm an advantage of our strategy, which we show in numerical simulations and SMLM experiments on a calibration sample containing specific “nanorulers” imaged via DNA paint [20] and of nuclear pore complexes (NPC) [21].

## 2. Motivation

**Fig. 1.**
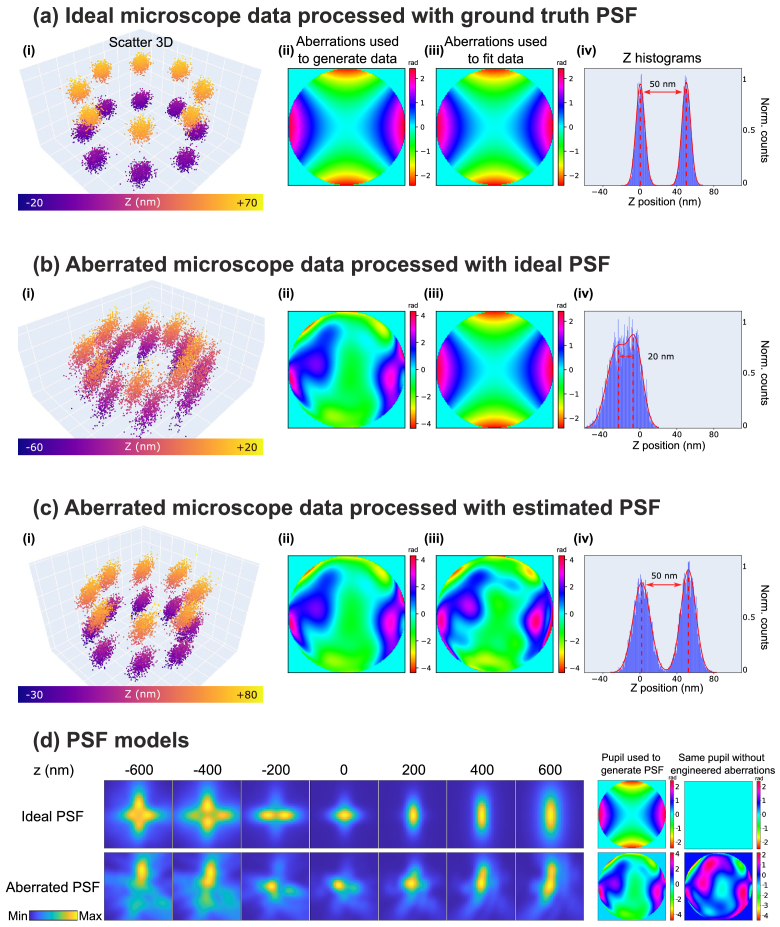
**Importance of knowing the PSF.** SMLM reconstructions of simulated image data of a structure resembling a Nuclear Pore Complex (NPC). We simulated astigmatic imaging (NA 1.49, emission wavelength $\lambda_{\text{em}}$ = 670 nm), with 5,000 signal and 20 background photons in each molecule image. **a**, ideal microscope data processed with ground truth PSF. **b**, aberrated microscope data processed with ideal PSF. **c**, aberrated microscope data processed with estimated PSF. The data for the reconstructions in (**i**) were generated with the aberrations in (**ii**) and fitted using the aberrations in (**iii**). The Z-histograms in (**iv**) show the estimated axial distance between the two ring structures. **d**, PSF models used in the previous points, with the respective phase aberrations. We also show the phase aberrations with the engineered part $Z_{6}=1$ rad subtracted.

Before we explain the details of our method, we would like to highlight and visualize the importance of reconstructing SMLM datasets under correct assumptions on the PSF. Figure 1 shows 3D object reconstructions from synthetic SMLM data sets. The assumed object resembles fluorescent molecules attached to an NPC. It consists of 16 molecules arranged in two circles of 120 nm diameter, which are axially separated by 50 nm. The synthetic SMLM data for this object consisted of $10^{4}$ widefield images, taken with an astigmatic PSF to improve the axial localization precision. Figure 1(a,ii) shows the pupil phase of the PSF that was used to generate the synthetic SMLM data. The saddle-shaped phase function, which corresponds to a primary Zernike astigmatism ( $Z_{6}$ ) of 1 rad magnitude, is clearly visible. A magnitude around 1 rad is about optimal for the chosen settings (NA = 1.49) [22]. Apart from the astigmatism, no additional phase distortions were added to the pupil phase, i.e., the PSF was assumed to be aberration free.

An SMLM reconstruction algorithm was applied to the images to produce a 3D image of the object. The algorithm fits a numerical PSF model to each single molecule image, delivering estimates of their 3D positions. In the first simulation, the algorithm used the same PSF for fitting the images as was used for constructing the synthetic data. As expected this leads to accurate results: Each molecule in the reconstruction shown in (a,i) is clearly resolved at an almost isotropic localization precision and the axial distance between the two NPC ring structures is accurately estimated (a,iv).

In a second simulation, the synthetic data was generated with an aberrated PSF, shown in (b,ii). The additional RMS aberration phase magnitude was 1 rad ( ≈λ/6 ), which is significant but may occur in real situations. If the reconstruction algorithm is wrongly assuming an aberration free PSF (b,iii), the data reconstruction appears visibly degraded (b,i) and the two rings appear at a wrong distance and remain axially unresolved (b,iv). Conversely, if the aberrations are known and taken into account in the reconstruction step, the image quality can be largely restored (c,i) and the rings appear resolved again at an accurate distance (c,iv). Of note, however, the localization precision cannot be restored, which explains why the result still falls short of the “ideal” reconstruction shown in (a,i). Full recovery of imaging performance requires the use of hardware AO such as a deformable mirror, which allows for the physical removal of wavefront aberrations. Lateral cross sections through the intensity PSF models at seven different z positions are shown in Fig. 1(d). The images on the right show the matching pupil phases, with (left) and without the engineered astigmatism. In this simulation, the aberrations shown in (c,iii) were retrieved by our reconstruction algorithm, jointly with the positions of all 16 molecules. In the following, we explain the working principle of our method in detail.

## 3. Method

**Fig. 2.**
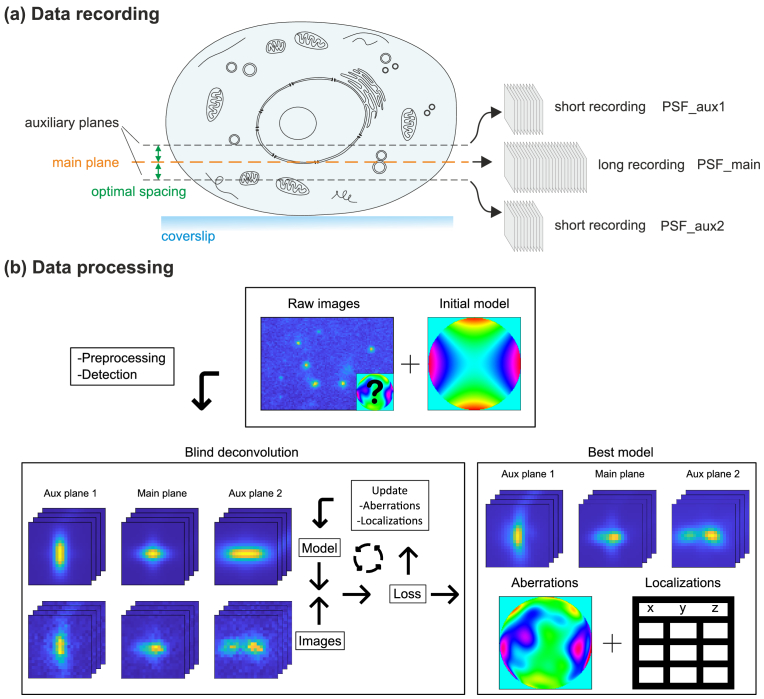
**Optimized blind deconvolution. a**, Data recording: The majority of SMLM images is collected with the focus set for optimal localization precision in the main plane. Additionally, the focal plane is moved to one or more auxiliary planes and 100 additional images are recorded at each. **b**, Data processing: raw images are converted into photons, cropped and fitted using an initial PSF model (here an astigmatic PSF) to discard bad images. Images at different planes are used to minimize the negative Log Likelihood Ratio via a gradient-based optimization algorithm, which jointly estimates aberrations and localizations.

Figure 2(a) outlines the workflow of our approach. The majority of SMLM data are acquired in a single plane containing most of the positional information about the molecules of interest, just as in any regular SMLM recording. The z-position of this main plane should be – together with the PSF shape – chosen to be optimal for this purpose, for instance, by maximizing the Fisher information (FI) with respect to the 3D position [22,23]. In addition to this main recording, short image tracks of the sample are taken at one or more auxiliary planes, whose z-positions are chosen to maximize information about aberrations. For a given NA and imaging conditions, it is possible to find optimal auxiliary plane positions using an analysis based on Fisher information. The search is related to that for optimal planes in multifocal plane microscopy [24], with the difference that the information about more than just one parameter, namely a set of about 10 - 20 Zernike mode magnitudes, must be maximized. A common approach to achieve this for general multi-parameter estimation problems is to maximize the determinant of the FI matrix. Such experimental settings are referred to as “D-optimal” [25], which indicates that the determinant of the FI matrix is optimized. Since the inverse of the FI matrix contains the parameter variances, maximizing the determinant of the FI matrix is equivalent to minimizing the determinant of the covariance matrix and hence the multidimensional “uncertainty volume”. Applied to our particular case, it means that we need to identify those z-planes for which the determinant of the total FI matrix, i.e., the sum of each plane’s FI matrix, with respect to all parameters of interest becomes maximal. For this work, these parameters include all Zernike mode magnitudes up to the fourth order (except piston), ranging from mode 5 (astigmatism) to mode 22 (secondary spherical) in the Noll index scheme [26]. Our analysis suggests that optimal planes should be located around 400 nm above and/or below the main imaging plane for high NA imaging (NA 1.5). The optimal plane distances increase with decreasing NA (calculations are provided in the Supplement 1).

We further find that taking data at these optimal planes also enhances the well-posedness of the inverse problem in general. When solving it using gradient-based optimization, the algorithm converges faster and is more likely to find the global optimum when fed with data from optimal planes compared to data from less optimal ones. Further information is provided in the Supplement 1.

After the recording step, the entire data, that is, the image series of all recorded planes, are fed to a gradient-based search algorithm implemented in the Python package JAX [27] to jointly estimate PSF shape and molecule positions. At its core lies a differentiable function that calculates a molecule image based on all parameters we want to fit: 3D position, signal and background level, as well as a set of coefficients defining a Zernike series to model phase aberrations in the objective pupil. Details about the implementation of this function are provided in the Supplement 1.

While 3D position, signal and background of each molecule are estimated individually, the aberration parameters are estimated collectively for an entire batch of molecules. This is due to the fact that aberrations are usually dependent on the position to a weak extent, which actually represents an important boundary condition for successfully solving the inverse problem. How much aberrations vary with position depends on the sample, if the sample itself causes them. For system aberrations, we observe no significant field dependence over a central field of view of more than 20 µm.

Notably, our algorithm allows the PSFs of each plane to be different, although they share the set of Zernike fit parameters to describe aberrations. This is necessary for TIRF microscopy, for example, where the 3D PSFs depend on the absolute distance of the emitter from the glass coverslip due to different contributions of supercritical angle fluorescence [28]. In this case, the PSF will change with the z-position, but aberrations will not. Another application example where the feature of plane-specific PSFs is necessary is the case of an RI mismatch between objective immersion medium and sample buffer, where a known spherical aberration term appears that increases in proportion with the distance of the molecule from the coverslip [29]. This spherical aberration term is already included in our numerical PSF model and therefore already considered when calculating the plane-specific PSFs. Conversely, any PSF engineering steps to improve the localization precision, for example the introduction of a cylindrical lens pair, *are* modeled by the Zernike fit parameters. This means that they only need to be approximately known to initialize the algorithm with a sufficiently close guess of the PSF.

### 3.1. Data processing

The forward model is represented by a function $I_{m,n}(a,v)$ , which calculates photon numbers for each pixel with index pair (m,n) in a single molecule image. The arguments of the forward function are vectors v=(x,y,z,s,BG) , which contains data about the molecule position x,y,z , background level BG in photons per pixel and signal photon number s , and a , which is a vector of Zernike coefficients. Details about the forward model are provided in the Supplement 1. Our algorithm minimizes the negative log-likelihood

(1) $$L(v_{1},\ldots ,v_{N_{img}},a)=\sum_{i=1}^{N_{img}}\sum_{m,n=1}^{N_{m},N_{n}}\left(I_{m,n}\left(v_{i},a\right)-M_{i,m,n}\log\left(I_{m,n}\left(v_{i},a\right)\right)\right)$$

where $M_{i,m,n}$ denotes the photon number in pixel with index pair (m,n) of the i -th measured molecule image and $N_{img}$ the total number of images. We minimize Eq. (1) jointly for $\left(v_{1},\ldots ,v_{N_{img}}\right)$ and a using gradient descent with Nesterov acceleration.

## 4. Results

### 4.1. Jointly retrieving PSF and localizations from a synthetic data set

We apply our method to synthetic SMLM data from a 3D structure resembling a nuclear pore complex (NPC) as shown in Fig. 1(a,i). This structure is challenging because its axial stretch is negligible compared to that of the PSF, resulting in a lack of the image diversity needed to render the inverse problem well solvable. The structure is assumed to be immersed in water and sitting directly on the glass coverslip. Experimental settings, such as NA and emission wavelength are the same as stated in section 2.

We tested our algorithm on a variety of synthetic data sets, each assuming different aberrations and signal to background ratios (SBR). In particular, we investigate the influence of the following parameters: i) total aberration magnitude, ii) SBR, iii) cumulative signal, i.e. the average number of photons detected from a single molecule multiplied with the number of imaged molecules, iv) number of auxiliary planes at which data are recorded. Obviously, the chosen imaging method will likewise have an effect. There exist a plethora of implementations for 3D SMLM, including astigmatic imaging [30,31], multifocal plane imaging [32], and defocused imaging [28,33], as well as numerous implementations utilizing more complex, engineered PSFs [23,34–40]. In this simulation study, we focus on the probably most prevalent method, namely astigmatic imaging, which is modeled by adding Zernike primary astigmatism with a magnitude of 1 rad, which is about optimal for our settings [22].

We assume aberrations of different magnitudes. The total aberration magnitude is quantified as the standard deviation of the emitted light phase in the objective pupil. Considering the definition of the diffraction limit, for which the standard deviation of the phase is $\sqrt{0.2}\approx 0.45$ rad according to the Maréchal criterion [41,42], we consider three different magnitudes: “mild” (0.5 rad), “strong” (1.0 rad), and “severe” (1.5 rad), and randomly distribute the total magnitude over the first 10 radial Zernike orders (up to Noll index 66), skipping piston, tilt, and defocus. Typically, lower index modes appear at higher magnitudes compared to Zernikes of higher index. Therefore, we skew the distribution towards lower index modes such that the distribution resembles typical results we obtain from phase retrieval measurements on microbeads [43]. Detailed information about the generation of aberrations for these simulations are provided in the Supplement 1.

Aside from aberrations, we expect the following two parameters of single molecule images to affect the performance of our algorithm: the signal-to-background ratio (SBR) and the total cumulative signal (avg. signal of molecule × number of detected emitters). In this work, signal s denotes the total number of photons emitted by a molecule and collected by the objective lens. Of note, the number of photons that end up in a single SMLM image is somewhat smaller, because not 100% of those photons end up in the tightly cropped molecule image and there is also some loss due to the assumed sCMOS detector with a Quantum Efficiency of 75% and a readout noise/pixel of 0.7 electrons. For each aberration setting, we repeated the simulation with different SBRs and cumulative signals. To quantify the goodness of the algorithm fits to the aberrations, we use the square root sum of the polynomial differences:

(2) $$\varepsilon rr=\sqrt{\sum_{i}{\left(a_{i}-{\hat{a}}_{i}\right)}^{2}},$$

where $a_{i}$ is the i-th magnitude of the ground truth (i.e., the assumed magnitude of the Zernike mode $Z_{i}$ ), and ${\hat{a}}_{i}$ the respective estimate. We noticed that the algorithm performs always well ( εrr < 0.05 rad) on synthetic data, regardless of the aberration magnitude, if identical sets of Zernike modes are assumed for both, the ground truth aberration and the fits. As realistic aberrations will not be restricted to a certain set of low-order Zernikes, we included modes up to the 10^th^ order (62 modes) for the generation of synthetic images, but fitted only modes up to the 5^th^ order (18 modes), including secondary spherical ( $Z_{22}$ ).

**Fig. 3.**
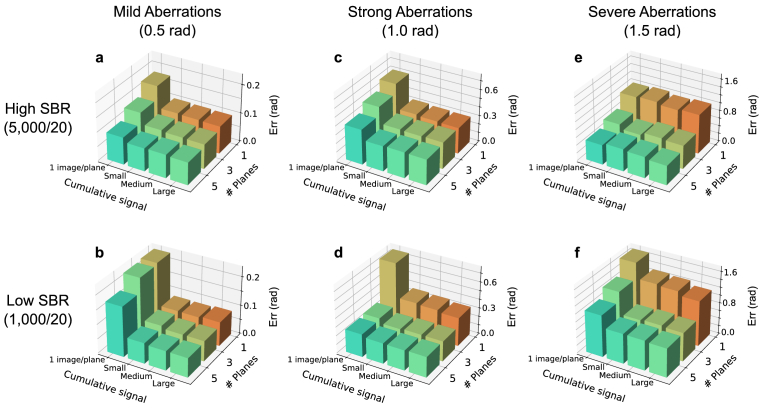
**Residual aberration errors when executing our fit algorithm for different parameter combinations.** The bars show the errors as defined in Eq. (2). Every bar is the average of three independent simulations. The aberrations are generated with three different magnitudes: Mild (0.5 rad) (**a**, **b**), strong (1.0 rad) (**c**, **d**), and severe (1.5 rad) (**e**, **f**). In the upper row (**a**, **c**, **e**) we assume a high SBR of 5,000/20 photons while in the lower row (**b**, **d**, **f**) we assume a low SBR of 1,000/20 photons. Every plot includes simulations for different cumulative signals (1 image/plane $\approx 10^{4}$ , small = $10^{5}$ , medium = $10^{6}$ , large = $10^{7}$ ), as well as for imaging at one, three and five planes, respectively.

Figure 3 summarizes our simulation results. Here we plot εrr for three different aberration magnitudes (0.5, 1.0, and 1.5 rad), each with high (5,000/20) and low (1,000/20) SBR. We also used three different recording plane configurations (1, 3 and 5 planes) and four different cumulative signal values ( $\approx 10^{4}$ , $10^{5}$ , $10^{6}$ , and $10^{7}$ ), resulting in a total of 12 bars per plot. 1 image/plane is the lowest cumulative signal possible in each simulation. It spans from a value of $10^{3}$ for the 1 plane deconvolution in the low SBR to a value of $2.5\times 10^{4}$ for a 5 plane deconvolution in a high SBR simulation. Each bar represents the average εrr of three independent runs of the algorithm, each assuming a different set of random aberrations. The simulations demonstrate good repeatability, with an average coefficient of variation ( ⟨σ/μ⟩ ) of 0.2, i.e. the standard deviation across runs is 20% of the mean value. This provides confidence in the robustness of the results.

The 1-plane strategy records molecule images only in the “main” plane, that is the long recording as sketched in Fig. 2(a), and should be maximally informative on the molecule positions. For astigmatic imaging, this focus plane should be roughly matching the average z-coordinate of the molecules, while for defocused imaging [28], the main focus plane is about $\lambda_{em}/2$ above the mean molecule plane [22]. The 3-planes strategy adds two auxiliary planes to the main plane, which are axially offset by ± 400 nm, and the 5-planes strategy adds two more at ± 200 nm. The cumulative signal parameter is controlled via the number of molecule images. Here, we span a wide range: For the smallest cumulative signal, we assume that only one image per plane is taken. The *small* cumulative signal equals $10^{5}$ photons, corresponding to 20 images for high SBR or 100 images for low SBR. *Medium* signal equals $10^{6}$ (200 and 1,000 images), and *large* is $10^{7}$ (2,000 and 10,000 images). These numbers are summarized in Table 1. The images are distributed evenly between planes and randomly between the two NPC layers.

**Table 1.** Parameters used for the simulations shown in Fig. 3. The cumulative signal is the product of the number of images with the average signal per image.

	Cumulative signal	# images high SBR	# images low SBR

1 image / plane	$\approx 10^{4}$	1 / plane	1 / plane
Small	$10^{5}$	20	100
Medium	$10^{6}$	200	1,000
Large	$10^{7}$	2,000	10,000

For mild aberrations [Fig. 3(a,b)], the algorithm works well for almost every combination of parameters, leaving only minimal residual aberrations below 0.1 rad. Noticeable differences in εrr between strategies involving different numbers of recording planes only occur for the smallest cumulative signal (only one molecule image per plane).

In the case of stronger aberrations, the multiplane strategies become more advantageous. Especially for severe aberrations, depicted in Fig. 3(e,f), the remaining errors of the 1 plane strategy are consistently larger than those achieved with the 3 and 5-plane strategies, even for the highest assumed cumulative signal and high SBR. The simulations further indicate that there is practically no advantage to using the 5-planes over the 3-planes strategy for any combination of aberration magnitudes, SBR and cumulative energies. This suggests that the additional information contributed by recordings taken at the middle auxiliary planes located at ± 200 nm is of negligible importance.

### 4.2. Experimental results

#### 4.2.1. DNA-PAINT imaging of nanorulers

We applied our method to DNA-PAINT data from commercial nanorulers (Gattaquant GATTA-PAINT 3D HiRes 80R) taken with an astigmatic PSF. The astigmatic PSF was shaped by a convex/concave cylindrical lens pair with focal lengths ±1,000 mm in the emission path. One of the lenses was rotated such that the net effect resulted in an astigmatic wavefront distortion of about 0.75 rad magnitude. The nanorulers consist of two spots labeled with ATTO 655 separated by 80 nm. They are attached to the coverslip on one side and show a broad angular orientation distribution with standing, tilted, and lying rulers. To test our algorithm, we deliberately misaligned the coverslip thickness correction collar of our objective lens (Olympus APON60XOTIRF) to introduce small spherical aberrations of ≈ 0.3 rad on top of the astigmatism. We took image data from five different focal planes located at the sample-coverslip interface and ± 200 and ± 400 nm around it. Each image shows an area of about 20 × 20 ${\mathrm{µm}}^{2}$ . 100 frames were taken at every plane, each with 100 ms exposure time. For each algorithm run, we used in total 100 images with an SBR of 1,500/40, resulting in a cumulative signal similar to the simulated "Small" value in Fig. 3.

**Fig. 4.**
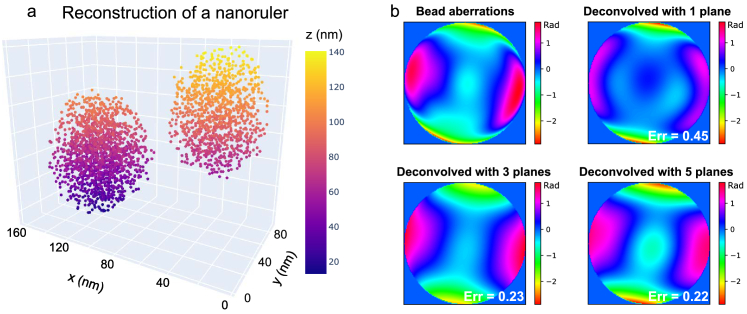
**Application of blind deconvolution to DNA-PAINT data taken with an astigmatic PSF. a**, SMLM image of a nanoruler, reconstructed jointly with pupil phase aberrations from images of 5 planes. The first pupil phase in **b** was measured from a high SNR bead stack, which is why we consider it as ground truth. The other pupil phases resulted from the joint estimation algorithm, using images taken in one plane, three planes ( ± 400 nm) and five planes ( ± 200 nm, ±400 nm). The errors stated in the phase images are in rad and referenced to the ground truth by Eq. (2). In this example, processing multi-plane data led to more accurate aberration estimates.

To assess the accuracy of the aberrations retrieved by our algorithm, we need a ground truth against which to compare them. For this purpose, we measured them in a separate step from a z-stack of a single fluorescent microbead on a coverslip [7]. The z-stack consists of 17 high SNR images ranging from ±1.6 µm around the bead, which is why this measurement can serve as a sufficiently reliable ground truth for our purpose. The software used for the bead phase retrieval is publicly available [43]. Figure 4 shows the estimated pupil phase aberrations. Apparently, all strategies were able to find the astigmatic aberrations, giving a square root error on the astigmatism modes (5 and 6) of 0.1 rad, while the single-plane strategy gives an error of ≈ 0.3 rad. The error on the spherical modes (11 and 22) is around 0.16 rad for all cases, with a slightly smaller error if data from 5 planes were processed. Despite the fact that the aberrations were more faithfully reconstructed by taking data from multiple planes, this had no noticeable effect on the the localization accuracy, i.e., the measured distance between the two dye molecules of single nanorulers did not change significantly.

#### 4.2.2. “Defocused imaging” 3D dSTORM of nuclear pore complexes

We applied our algorithm to SMLM image data taken from nuclear pore complexes (NPCs) [17,21] using a TIRF objective (Olympus UPLAPO100XOHR). The data were acquired from U2Os cells expressing Nup96-Snap-tag, labeled with Alexa Fluor 647 (AF647) and imaged with dSTORM [44] in a defocused imaging configuration [22,28]. The lower part of the nuclear membrane was in a close proximity to the coverslip and the NPCs were located in a range of 100-400 nm above. Of note, for defocused imaging, the main imaging plane is located about 300 nm above the NPCs (here set to be about 700 nm above the coverslip), such that the molecules appear out of focus. The auxiliary planes are both below this main plane in order to capture information about aberrations optimally, i.e., the aux. planes are located at +300 and −100 nm with respect to the coverslip, . The plane positions for the 5-planes strategy are then −100, +100, +300, +500, +700 nm with respect to the coverslip. The main stack comprised 50,000 frames and each auxiliary stack 1,000 frames. The exposure time for each image is 20 ms.

**Fig. 5.**
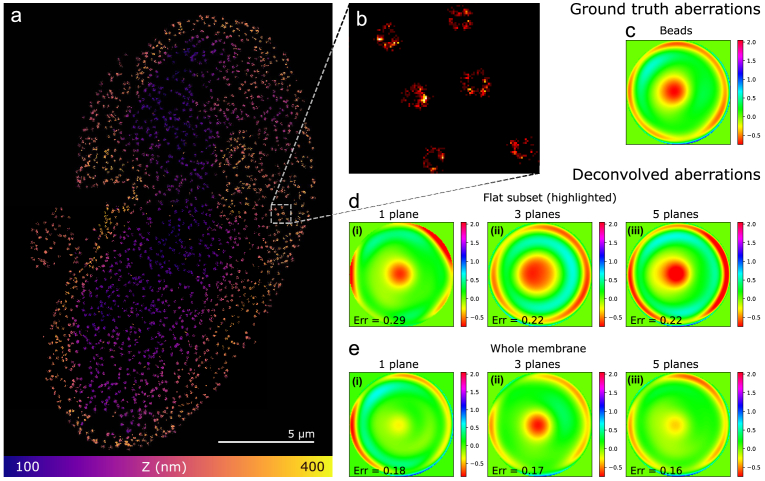
**Application of blind deconvolution to “defocused imaging” dSTORM near the coverslip. a**, reconstruction of NPCs in the lower cell membrane lying on the coverslip, labeled with AF-647. **b**, subset of NPCs with a small z-variation ( ≈ 70 nm). SMLM images from this region have negligible phase diversity and were used to determine the aberrations in **d**. The pupil **c** is derived from a bead stack and we consider it here as the ground truth. **d**, we used 100 SMLM images from **b** to retrieve these aberrations, while for the aberrations in **e** we used 2,000 SMLM images of molecules spread over the whole sample. The “1 plane” results are based on data from the plane 300 nm above the coverslip, the “3 planes” results on data that included the planes at ± 400 nm from the main plane, and the “5 planes” results on data that included the ± 200 nm planes as well. The error is in rad and is referenced to the ground truth by Eq. (2). To reconstruct **a** we used the “3-planes” aberrations in **d**;

Figure 5(c) shows the phase aberration in the objective pupil, measured from a single fluorescent bead, which defines our ground truth. The approximate radial symmetry indicates that the aberration predominantly consists of spherical modes (described for example by the Zernike Noll indices 11, 22, 37, and 56). Consequently, polynomials 37 and 56 were also included in the Zernike vector, resulting in the estimated Zernike coefficients $a=({\text{a}}_{5-22},{\text{a}}_{37},{\text{a}}_{56})$ .

We evaluate the performance of our method on two different data sets. The first set consists of 2,000 SMLM images with a SBR of ≈ 1,200/30 from the entire lower nuclear membrane (Fig. 5(a)), which fall close to the "low SBR" and “medium” category for the cumulative signal, as defined in Fig. 3. In this context, “lower” means the membrane in close proximity to the coverslip. Due to the non-flat topography of the nuclear membrane the molecules in this dataset cover an axial range of about 300 nm. The second set consists of 100 SMLM images from a small subregion within the nuclear membrane (Fig. 5(b)), containing only six NPCs located in the same optical plane (thickness ≈70 nm). The intrinsic lack of z-diversity among these images makes deconvolution particularly challenging. This dataset falls close to the “low SBR” and "small" cumulative signal category as defined in Fig. 3.

The pupil phase patterns recovered by our algorithm for both datasets are shown in Fig. 5(d,e). All patterns resemble the ground truth, demonstrating the effectiveness of the blind deconvolution approach in general. On closer inspection, when applied to the smaller, “flat” dataset of the subregion, we find that the pupil phase estimate of the multi-plane strategies (d,ii and d,iii) are closer to the ground truth than that of to the single-plane approach (d,i), which is consistent with the results of the nanorulers experiment. For the large dataset comprising the whole membrane, this improvement is not significant, i.e. the single-plane and multi-plane retrieval procedures deliver pupil phases with a comparable ground truth similarity (e). We attribute this to the larger image diversity and cumulative signal of the second set. Of note, as for the nanoruler experiment, the higher accuracy of the aberration estimates produced by the multi-plane procedures has no quantitative impact on the accuracy of the NPC reconstructions.

**Fig. 6.**
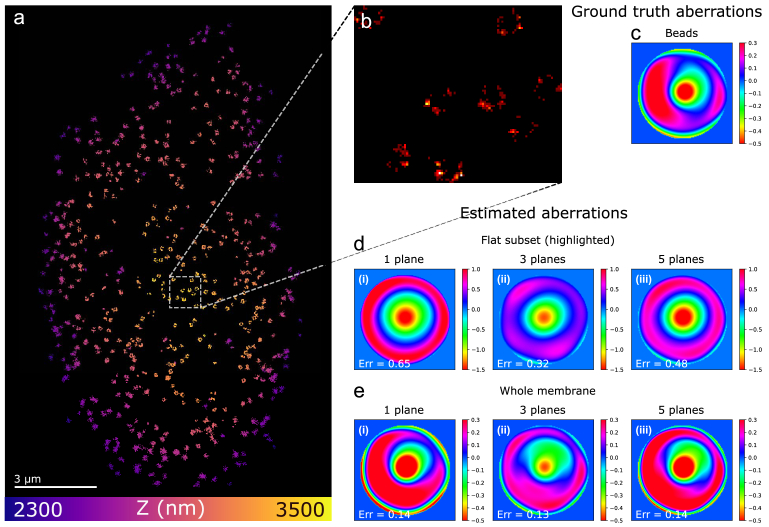
**Application of blind deconvolution to “defocused imaging” dSTORM far from the coverslip. a**, reconstruction of NPCs in the upper cell membrane labeled with AF-647. The objective is focused 4 µm above the coverslip. **b**, subset of NPCs with a small z-variation ( ≈ 70 nm). SMLM images from this region have negligible phase diversity and were used to determine the aberrations in **d**. **c**, bead aberrations restricted to the UAF emission given by the sample RI of 1.35. We consider it here as the ground truth. **d**, we used 100 SMLM images from **b** to retrieve these aberrations, while for the aberrations in **e** we used 2,000 SMLM images spread over the whole sample. The “1 plane” results are based on data from the main image plane at about 4 µm above the coverslip, the “3 planes” results on data that included the planes at ± 800 nm from the main plane, and the “5 planes” results on data that included the ± 400 nm planes as well. The error is in rad and is referenced to the ground truth by Eq. (2). To reconstruct **a** we used the “3-planes” result in **d**.

We repeated the same measurements and analysis on the upper nuclear membrane, which poses additional challenges: Firstly, due to the large distance of several micrometers from the coverslip, there is no supercritical angle fluorescence (SAF) emission, which reduces the signal and NA. Secondly, the RI mismatch between the cell nucleus and immersion oil introduces spherical aberrations on the order of 0.1 µm RMS ( ≈ 1 rad at 670 nm wavelength), if we assume ${\text{RI}}_{\text{nucleus}}$ = 1.35 and a nucleus thickness of 4 µm. For measuring the upper membrane we focused the objective to 4 µm above the coverslip. When processing the data, we assumed the same ground truth pupil phase aberrations as for the measurement on the lower nuclear membrane, because the additional spherical aberrations introduced by the RI mismatch are already included in our molecule image model. This means that the ground truth phases of Figs. 5(c) and 6(c) are essentially identical, except for the smaller NA in the latter image, which was set to the RI of the buffer medium ( ${\text{RI}}_{\text{buffer}}=1.35$ ). In general, the effective pupil radius in k-space is given by: $\text{k}={\text{k}}_{\text{r}}\cdot ({\text{RI}}_{\text{sample}}/{\text{RI}}_{\text{glass}})$ , where ${\text{k}}_{\text{r}}$ is the TIRF radius $\text{NA}\cdot 2\pi /\lambda_{em}$ .

In this experiment, we again processed the full data set comprising the entire membrane as well as data from a “flat” subset, which produced different aberration results. While that obtained from the full data set resembles the ground truth, probably because the molecules shows a large enough spread along z, the aberrations obtained from the flat subset show an additional contribution of about −0.4 rad primary spherical (Noll mode 11). We speculate that this difference is not an artifact, but may at least in part reflect different local aberrations or even differences in the mean RI of the regions covered by the respective emission light cones. Bearing in mind that these cones have very large aperture angles within the sample, we find that the light emitted by the dye molecules in the central sub-region propagates predominantly through the nucleus, whereas that of the molecules located at the edge zones propagates predominantly through the buffer or other cell compartments, which have a different RI. Indeed, for some investigated cell types the material inside cellular nuclei has been found to have a lower RI [45]. However, the expected difference is on a small scale ( ≈0.01−0.02 ) and a more detailed analysis reveals that this cannot fully explain our observation. If we attributed the change of $\Delta Z_{11}=-0.4$ rad exclusively to a different RI of the medium underneath the emitting molecules, it turns out that the medium would need to have an RI of 1.32, i.e. lower than that of water. This shows that the aberration difference must predominantly stem from other sources. It could also partly be explained by fit errors, which are expected to lie on the order of 0.1 rad.

To check the impact of the different aberration results on the localization bias, we took a closer look at the respective estimates for the axial distance of the two 8-fold NPC rings. As this value is known to be 50 nm, any systematic deviation will reveal an incorrect model of the molecular image.

**Fig. 7.**
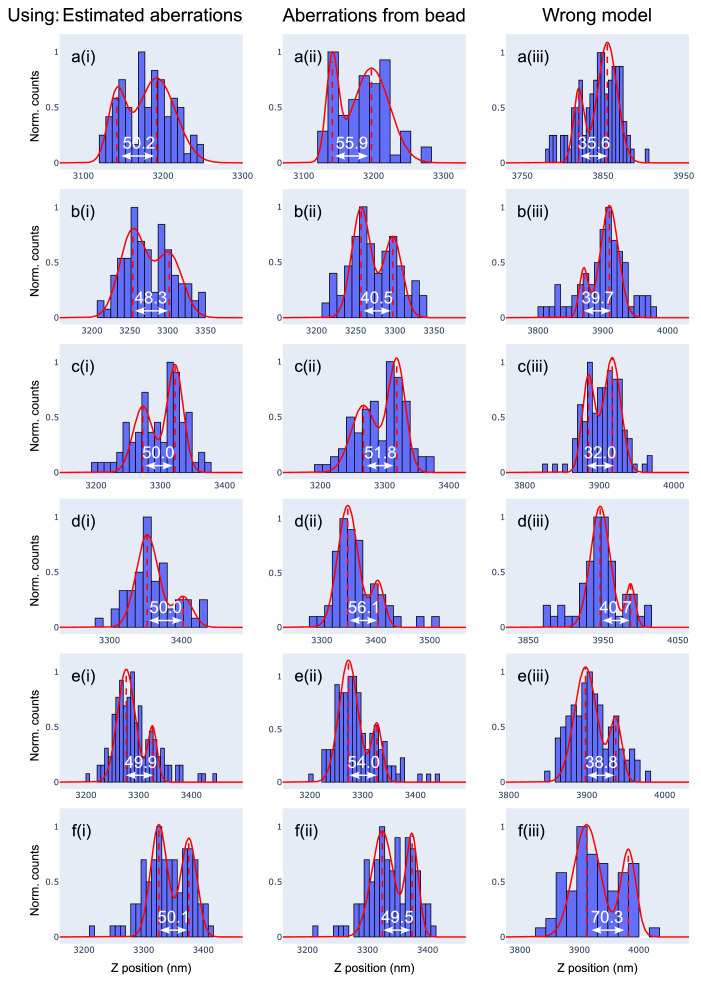
**Comparison of PSF models.** z-localization histograms of six NPCs in the upper membrane, close to the subregion shown in Fig. 6(b). Each row shows the same NPC, reconstructed using three aberration models, which correspond to the columns. Model (i) uses the aberrations retrieved from SMLM images in the flat region (Fig. 6(d)(ii)). Model (ii) uses aberrations derived from a bead on the coverslip (Fig. 6(c)). Model (iii) also applies the aberrations from (ii) but assumes a sample refractive index of 1.52, neglecting the RI mismatch effect. Consequently, model (iii) produces inaccurate z localizations, unlike models (i) and (ii), which account for this effect.

Figure 7 shows results of this investigation in the form of histograms of molecule z positions. Each row represents data from one of six NPCs that are are either inside or very close (<1 µm) to the rectangular subregion of Fig. 6(b) and each NPC has been processed assuming three different aberration models, which correspond to the columns. For the model in the first column we used the aberrations retrieved from 100 images in 3 planes, shown in Fig. 6(d)(ii), assuming ${\text{RI}}_{\text{buffer}}=1.35$ . In the second column we used the bead aberrations (Fig. 6(c)), again assuming ${\text{RI}}_{\text{buffer}}=1.35$ . For comparison we added a third column showing NPC data constructed with an apparently wrong model. Here, the same bead aberrations have been used as in the second column, but the sample RI has been set to the value of the immersion oil, i.e., the RI mismatch has been ignored: ${\text{RI}}_{\text{buffer}}=1.52$ . Our motivation for including this third column is to show the magnitude of the systematic errors which arise when a z-stack of a fluorescent bead on the coverslip is directly interpreted as the imaging PSF.

The results in the first two columns show very similar NPC layer separations of 49.75(0.66) nm when using the aberrations retrieved by our algorithm (1st column) and 51.30(5.35) nm when using the bead aberrations. Despite the small difference between these results, the fact that using the retrieved aberrations provides a more accurate ring separation with a smaller spread may indicate that the additional spherical aberrations retrieved by our algorithm do indeed provide a better molecular image model for the small subregion on the upper nuclear membrane.

With regard to the question of whether processing data from multiple image planes offers any advantage over the use of a single plane, the experiment presented in Fig. 6 provides only limited insight. This is because, under the specified SBR conditions, the one-plane approach proved to be sufficient. It is noteworthy that this outcome aligns with the findings of our simulations on synthetic data (Fig. 3).

## 5. Discussion

This work represents an investigation into the feasibility and robustness of blind deconvolution techniques in super-resolution microscopy (SMLM). By jointly estimating molecule positions and pupil phase aberrations, such techniques can prevent biases caused by errors in PSF shape assumptions. They promise not only to render separate calibration steps using micro beads redundant, but also to facilitate the identification and incorporation of field-dependent aberrations introduced by the optics and sample. For these reasons, blind deconvolution techniques can be a valuable asset in increasing the reliability of SMLM images.

We hypothesize that blind deconvolution strategies can be ill-posed when imaging "flat" samples stretching only about 100 nm or less along the optical axis. In these cases, the recorded molecule images show only little diversity, making it difficult to reliably separate the impact of the z position parameter and spherical aberrations. We further hypothesize that taking SMLM data from multiple axial planes should increase the image diversity and, therefore, improve the robustness of blind deconvolution.

Based on the second hypothesis, we propose a simple strategy to increase the robustness of blind deconvolution in SMLM. This strategy involves recording and processing additional, short image tracks at two or more auxiliary planes. We identify optimal positions for these planes using a numerical optimization based on Fisher information, finding that ±400 nm around the central image plane are about optimal for high NA imaging (NA 1.49).

We present a detailed numerical investigation that confirms our hypotheses. Specifically, we identify imaging conditions where blind deconvolution in SMLM is prone to fail and observe that even strong aberrations of around 1 rad magnitude should still be identifiable as long as the SBR and accumulated photon numbers are sufficiently high. For very strong aberrations (1.5 rad or more), blind deconvolution fails irrespective of the SBR and signal quality.

Experimental SMLM data from DNA-PAINT calibration samples and nuclear pore complexes demonstrate, at least under poor imaging conditions, the advantages of the multi-plane strategy. Notably, aberration estimates obtained from multi-plane data are frequently closer to the ground truth than those derived from single-plane data. However, 1-plane blind deconvolution was rarely observed to fail entirely, and discrepancies in aberration estimates obtained using single- or multi-plane approaches did not significantly affect SMLM data reconstructions. Instead, the algorithm performance was observed to be more reliable and less sensitive to parameter settings (e.g., learning rates) when processing data from multiple planes.

## 6. Conclusion and outlook

From the numerical studies and experiments conducted, it can be concluded that the proposed multi-plane strategy is generally advantageous, though it becomes strictly necessary only under unfavorable imaging conditions, such as settings with very low SBRs or small cumulative signals.

We have implemented a GPU-accelerated algorithm using the Python package JAX that is capable of processing SMLM data from multiple subsets (i.e. planes). Since our implementation supports the definition of individual PSFs for each subset, it can be extended to generalize the phase diversity concept beyond the use of data from different axial planes. For example, a more general "multi-view" approach could use a deformable mirror to produce a more informative PSF variation between the data subsets.

Of note, related approaches are regularly used in adaptive optics for microscopy, where many images taken at different applied aberration biases allow for robust aberration estimates [46]. A more integrated phase diversity approach, where object and aberrations are jointly estimated, has recently been reported in widefield fluorescence microscopy [47] and may represent a promising future direction for blind deconvolution in SMLM.

Finally, the introduction of hardware-based AO, such as a deformable mirror, would further allow to compensate for the intrinsic shortcomings of “digital AO” approaches as presented here, which are problems in faithfully recovering very strong aberrations and the lack of ability to improve localization precision.

## Supplemental information

Supplement 1 Supplement 1 https://doi.org/10.6084/m9.figshare.28435136

## Data Availability

Data underlying the results presented in this paper are not publicly available at this time but may be obtained from the authors upon reasonable request.

## Supplemental document

See Supplement 1 for supporting content.

## References

[1] Lelek M. Gyparaki M. T. Beliu G. et al., “Single-molecule localization microscopy,” Nat. Rev. Methods Primers 1(1), 39 (2021). 10.1038/s43586-021-00038-x 35663461 PMC9160414

[2] Hanser B. M. Gustafsson M. G. Agard D. et al., “Phase-retrieved pupil functions in wide-field fluorescence microscopy,” J. Microsc. 216(1), 32–48 (2004). 10.1111/j.0022-2720.2004.01393.x 15369481

[3] Quirin S. Pavani S. R. P. Piestun R., “Optimal 3d single-molecule localization for superresolution microscopy with aberrations and engineered point spread functions,” Proc. Natl. Acad. Sci. 109(3), 675–679 (2012). 10.1073/pnas.1109011108 22210112 PMC3271897

[4] Liu S. Kromann E. B. Krueger W. D. et al., “Three dimensional single molecule localization using a phase retrieved pupil function,” Opt. Express 21(24), 29462–29487 (2013). 10.1364/OE.21.029462 24514501 PMC3867195

[5] Petrov P. N. Shechtman Y. Moerner W., “Measurement-based estimation of global pupil functions in 3d localization microscopy,” Opt. Express 25(7), 7945–7959 (2017). 10.1364/OE.25.007945 28380911 PMC5810908

[6] Siemons M. Hulleman C. Thorsen R. et al., “High precision wavefront control in point spread function engineering for single emitter localization,” Opt. Express 26(7), 8397–8416 (2018). 10.1364/OE.26.008397 29715807

[7] Zelger P. Kaser K. Rossboth B. et al., “Three-dimensional localization microscopy using deep learning,” Opt. Express 26(25), 33166–33179 (2018). 10.1364/OE.26.033166 30645473

[8] Hieu Thao N. Soloviev O. Verhaegen M., “Phase retrieval based on the vectorial model of point spread function,” J. Opt. Soc. Am. A 37(1), 16–26 (2020). 10.1364/JOSAA.37.000016 32118876

[9] Ferdman B. Nehme E. Weiss L. E. et al., “Vipr: vectorial implementation of phase retrieval for fast and accurate microscopic pixel-wise pupil estimation,” Opt. Express 28(7), 10179–10198 (2020). 10.1364/OE.388248 32225609

[10] Li Y. Mund M. Hoess P. et al., “Real-time 3d single-molecule localization using experimental point spread functions,” Nat. Methods 15(5), 367–369 (2018). 10.1038/nmeth.4661 29630062 PMC6009849

[11] Möckl L. Petrov P. N. Moerner W., “Accurate phase retrieval of complex 3d point spread functions with deep residual neural networks,” Appl. Phys. Lett. 115(25), 251106 (2019). 10.1063/1.5125252 32127719 PMC7043838

[12] Gutiérrez-Cuevas R. Alemán-Casta neda L. A. Herrera I. et al., “Vectorial phase retrieval in super-resolution polarization microscopy,” APL Photonics 9(2), 026106 (2024). 10.1063/5.0179906

[13] Chung K. K. Baddeley D., “Estimating the psf from single molecule data,” Biophys. J. 112(3), 143a–144a (2017). 10.1016/j.bpj.2016.11.78928076805

[14] Xu F. Ma D. MacPherson K. P. et al., “Three-dimensional nanoscopy of whole cells and tissues with in situ point spread function retrieval,” Nat. Methods 17(5), 531–540 (2020). 10.1038/s41592-020-0816-x 32371980 PMC7289454

[15] Fazel M. Kilic Z. Nevskyi O. et al., “Simultaneous single particle tracking, phase retrieval and psf reconstruction,” Biophys. J. 122(3), 280a (2023). 10.1016/j.bpj.2022.11.1593

[16] Zhang P. Ma D. Cheng X. et al., “Deep learning driven adaptive optics for single molecule localization microscopy,” Biophys. J. 122(3), 430a (2023). 10.1016/j.bpj.2022.11.2328 PMC10630144 37770712

[17] Liu S. Chen J. Hellgoth J. et al., “Universal inverse modeling of point spread functions for smlm localization and microscope characterization,” Nat. Methods 21(6), 1082–1093 (2024). 10.1038/s41592-024-02282-x 38831208 PMC12330227

[18] Gonsalves R. A., “Phase retrieval and diversity in adaptive optics,” Opt. Eng. 21(5), 829–832 (1982). 10.1117/12.7972989

[19] Paxman R. G. Schulz T. J. Fienup J. R., “Joint estimation of object and aberrations by using phase diversity,” J. Opt. Soc. Am. A 9(7), 1072–1085 (1992). 10.1364/JOSAA.9.001072

[20] Schmied J. J. Forthmann C. Pibiri E. et al., “Dna origami nanopillars as standards for three-dimensional superresolution microscopy,” Nano Lett. 13(2), 781–785 (2013). 10.1021/nl304492y 23362960

[21] Thevathasan J. V. Kahnwald M. Cieśliński K. et al., “Nuclear pores as versatile reference standards for quantitative superresolution microscopy,” Nat. Methods 16(10), 1045–1053 (2019). 10.1038/s41592-019-0574-9 31562488 PMC6768092

[22] Zelger P. Bodner L. Offterdinger M. et al., “Three-dimensional single molecule localization close to the coverslip: a comparison of methods exploiting supercritical angle fluorescence,” Biomed. Opt. Express 12(2), 802–822 (2021). 10.1364/BOE.413018 33680543 PMC7901312

[23] Aristov A. Lelandais B. Rensen E. et al., “Zola-3d allows flexible 3d localization microscopy over an adjustable axial range,” Nat. Commun. 9(1), 2409 (2018). 10.1038/s41467-018-04709-4 29921892 PMC6008307

[24] Tahmasbi A. Ram S. Chao J. et al., “Designing the focal plane spacing for multifocal plane microscopy,” Opt. Express 22(14), 16706–16721 (2014). 10.1364/OE.22.016706 25090489 PMC4162350

[25] Atkinson A. Donev A. Tobias R., *Optimum Experimental Designs, with SAS* , vol. 34 (OUP Oxford, 2007).

[26] Noll R. J., “Zernike polynomials and atmospheric turbulence,” J. Opt. Soc. Am. 66(3), 207–211 (1976). 10.1364/JOSA.66.000207

[27] Bradbury J. Frostig R. Hawkins P. et al., “JAX: composable transformations of Python+NumPy programs,” (2018).

[28] Zelger P. Bodner L. Velas L. et al., “Defocused imaging exploits supercritical-angle fluorescence emission for precise axial single molecule localization microscopy,” Biomed. Opt. Express 11(2), 775–790 (2020). 10.1364/BOE.375678 32206395 PMC7041438

[29] Booth M. J. Neil M. A. Wilson T., “Aberration correction for confocal imaging in refractive-index-mismatched media,” J. Microsc. 192(2), 90–98 (1998). 10.1111/j.1365-2818.1998.99999.x

[30] Kao H. P. Verkman A., “Tracking of single fluorescent particles in three dimensions: use of cylindrical optics to encode particle position,” Biophys. J. 67(3), 1291–1300 (1994). 10.1016/S0006-3495(94)80601-0 7811944 PMC1225486

[31] Huang B. Wang W. Bates M. et al., “Three-dimensional super-resolution imaging by stochastic optical reconstruction microscopy,” Science 319(5864), 810–813 (2008). 10.1126/science.1153529 18174397 PMC2633023

[32] Juette M. F. Gould T. J. Lessard M. D. et al., “Three-dimensional sub–100 nm resolution fluorescence microscopy of thick samples,” Nat. Methods 5(6), 527–529 (2008). 10.1038/nmeth.1211 18469823

[33] Speidel M. Jonáš A. Florin E.-L., “Three-dimensional tracking of fluorescent nanoparticles with subnanometer precision by use of off-focus imaging,” Opt. Lett. 28(2), 69–71 (2003). 10.1364/OL.28.000069 12656488

[34] Baddeley D. Cannell M. B. Soeller C., “Three-dimensional sub-100 nm super-resolution imaging of biological samples using a phase ramp in the objective pupil,” Nano Res. 4(6), 589–598 (2011). 10.1007/s12274-011-0115-z

[35] Pavani S. R. P. Piestun R., “Three dimensional tracking of fluorescent microparticles using a photon-limited double-helix response system,” Opt. Express 16(26), 22048–22057 (2008). 10.1364/OE.16.022048 19104639

[36] Lew M. D. Lee S. F. Badieirostami M. et al., “Corkscrew point spread function for far-field three-dimensional nanoscale localization of pointlike objects,” Opt. Lett. 36(2), 202–204 (2011). 10.1364/OL.36.000202 21263500 PMC3196662

[37] Prasad S., “Rotating point spread function via pupil-phase engineering,” Opt. Lett. 38(4), 585–587 (2013). 10.1364/OL.38.000585 23455144

[38] Jia S. Vaughan J. C. Zhuang X., “Isotropic three-dimensional super-resolution imaging with a self-bending point spread function,” Nat. Photonics 8(4), 302–306 (2014). 10.1038/nphoton.2014.13 25383090 PMC4224117

[39] Shechtman Y. Sahl S. J. Backer A. S. et al., “Optimal point spread function design for 3d imaging,” Phys. Rev. Lett. 113(13), 133902 (2014). 10.1103/PhysRevLett.113.133902 25302889 PMC4381866

[40] Shechtman Y. Weiss L. E. Backer A. S. et al., “Precise three-dimensional scan-free multiple-particle tracking over large axial ranges with tetrapod point spread functions,” Nano Lett. 15(6), 4194–4199 (2015). 10.1021/acs.nanolett.5b01396 25939423 PMC4462996

[41] Born M. Wolf E., “Principles of optics. sixth (corrected) edition,” (1997).

[42] Maréchal A., *Étude des effets combinés de la diffraction et des aberrations géométriques sur l’image d’un point lumineux …* (Éditions de la Revue d’optique théorique et instrumentale, 1948).

[43] Schneider M. C. Hinterer F. Jesacher A. et al., “Interactive simulation and visualization of point spread functions in single molecule imaging,” Opt. Commun. 560, 130463 (2024). 10.1016/j.optcom.2024.130463

[44] Heilemann M. Van De Linde S. Schüttpelz M. et al., “Subdiffraction-resolution fluorescence imaging with conventional fluorescent probes,” Angew Chem. Int. Ed. 47(33), 6172–6176 (2008). 10.1002/anie.200802376 18646237

[45] Schürmann M. Scholze J. Müller P. et al., “Cell nuclei have lower refractive index and mass density than cytoplasm,” J. Biophotonics 9(10), 1068–1076 (2016). 10.1002/jbio.201500273 27010098

[46] Neil M. A. Booth M. J. Wilson T., “New modal wave-front sensor: a theoretical analysis,” J. Opt. Soc. Am. A 17(6), 1098–1107 (2000). 10.1364/JOSAA.17.001098 10850481

[47] Johnson C. Guo M. Schneider M. C. et al., “Phase-diversity-based wavefront sensing for fluorescence microscopy,” Optica 11(6), 806–820 (2024). 10.1364/OPTICA.518559

