# Supplementary material for: Joint estimation of point spread function and molecule positions in SMLM informed from multiple planes
Source: Biomed Opt Express. 2025 Mar 4;16(4):1310–26. doi: 10.1364/BOE.551278 (PMC12047720; doi:10.1364/BOE.551278)
Supplement: Supplementary file 1 [file boe-16-4-1310-s001.pdf]

Supplemental Document

Biomedical Optics EXPRESS

Joint estimation of point spread function and molecule positions in SMLM informed from multiple planes: supplement

JULIAN G. MALOBERTI,[1] LUKAS VELAS,[2] SIMON MOSER,[1,*] ANNA GAUGUTZ,[2] MARINA BISHARA,[2] GERHARD J. SCHÜTZ,[2] AND ALEXANDER JESACHER[1]

[1] *Institute of Biomedical Physics, Medical University of Innsbruck, Müllerstraße 44, 6020 Innsbruck, Austria*
[2] *Institute of Applied Physics, TU Wien, Getreidemarkt 9, 1060 Vienna, Austria*
[*] *simon.moser@i-med.ac.at*

This supplement published with Optica Publishing Group on 4 March 2025 by The Authors under the terms of the Creative Commons Attribution 4.0 License in the format provided by the authors and unedited. Further distribution of this work must maintain attribution to the author(s) and the published article's title, journal citation, and DOI.

Supplement DOI: https://doi.org/10.6084/m9.figshare.28435136

Parent Article DOI: https://doi.org/10.1364/BOE.551278

Joint Estimation of Point Spread Function and Molecule Positions in SMLM informed from multiple planes: supplemental document

This supplemental document contains additional information about our work on jointly estimating molecule positions and aberrations from SMLM data.

1. PSF CALCULATIONS

Our PSF calculations are based on the vectorial PSF model by Axelrod [1], which delivers a 2D molecule image based on the following input parameters: i) Mean emission vacuum wavelength λ_{em}. ii) Refractive indices of buffer solution, coverglass and an optional dielectric and transparent layer on top of the coverglass, which are summarized in a vector **RI**. iii) Thickness d_{layer} of the optional transparent layer. iv) Spatial distance z of the molecule from the coverslip. (v) Orientation (ϕ, θ) of the molecule. vi) Desired effective pixel size P of the simulated focal plane grid. We extended the original Axelrod model by adding signal s and background level BG, a defocus value Γ describing the relative position of the objective lens with respect to the coverglass and an aberration vector $\mathbf{a}$, which contains the Zernike mode magnitudes (according to the single-indexed Noll scheme) modeling phase aberrations in the objective pupil.

Because fluorescent markers in aqueous media at room temperature are usually “fast tumblers” that can be assumed to have isotropic emission characteristics, we calculate three images of molecules with mutually orthogonal emission dipole orientations and add their intensities in the focal plane to obtain the intensity image of a single dye molecule.

We further account for modeling errors arising from the fact that physical camera pixels are integrating photoelectrons over their square areas, while calculated molecule images consist of values at discrete points (the respective center points of the pixels). To mitigate this error, we calculate the PSF models at a higher spatial resolution than provided by the camera, and subsequently bin adjacent pixels to super-pixels that match the camera pixel size. We find that binning 2×2 pixels is usually sufficient for our effective pixel size $P = 160$ nm.

Finally, our model further includes a recently described blurring effect of back-illuminated cameras [2]. The effect originates from the travel of photoelectrons through the relatively thick silicon layer, which can sometimes drift sideways and end in adjacent pixels, effectively causing pixel-crosstalk. We model this effect by applying a Gaussian blur kernel (σ = 80 nm) to the molecule image.

All the described steps result in a numerical model for the PSF of the microscope, that is a model for the expectancy values of photon numbers collected in the camera pixels with indices (m,n): $h_{m,n}(x, y, z)$, where x, y, z are the 3D coordinates of a single molecule in the focal space. We finally normalize each x-y-slice in h to an integrated value of 1 over the entire simulated focal plane, which typically measures about 10×10 µm^2 (64×64 pixels at an effective pixel size of 160 nm).

2. GENERATION OF SYNTHETIC SMLM IMAGES OF NUCLEAR PORE COMPLEXES

To evaluate and validate our algorithms, we generated realistic synthetic SMLM image stacks mimicking the structure of Nuclear Pore Complexes (NPCs). NPCs are macromolecular assemblies consisting of two circular layers axially separated by 50 nm. Each layer has a radius of ≈60 nm and contains eight fluorescent molecules arranged in a symmetric circular pattern.

The synthetic image generation process involves simulating fluorescence emission from these NPCs while incorporating realistic experimental factors such as molecule blinking, background noise, and camera characteristics. Molecules are randomly toggled between an "on" state, where they emit photons, and an "off" state, reflecting the stochastic nature of single-molecule fluores-

cence. Each molecule has a probability of 10% to be "on" in any given frame, creating a dynamic dataset that mimics real experimental conditions.

Each molecule of the NPC has an assigned x,y,z position in focal space and an assigned number of photons that are emitted and collected by the objective (the signal s). Adding a constant background light level BG results in a mathematical expression for the expected numbers of photons arriving at every pixel in the camera plane. Finally, to arrive at a single simulated camera image in units of digital counts, we calculate Poissonian random numbers based on these expectancy values (function Poiss() in the equation below) and consider camera parameters such as the quantum efficiency QE (the number of created photoelectrons per incident photon), gain value G (the ratio of created photoelectrons over the digital count number) and the additive camera baseline value B. Before the photo-electron numbers are converted to digital counts, Gaussian noise N_{cam} is added to mimic the sensor readout noise. Together this results in a simulated camera image C:

$$C_{m,n} = \frac{1}{G}\left(\text{QE} \cdot \text{Poiss}\left[\text{BG} + s \cdot h_{m,n}(x,y,z)\right] + N_{\text{cam}}\right) + B \tag{S1}$$

Before images $C_{m,n}$ are processed with our algorithm (simulated and experimental ones), the baseline value B is subtracted and the result is multiplied by G/QE to obtain the quantity $I_{m,n}$ described in section 3.1 of the main document.

3. CALCULATION OF ABERRATIONS FOR THE SIMULATIONS IN SECTION 4.1. OF THE MAIN DOCUMENT

We use the following approach to prepare phase aberrations for synthetic SMLM data: after generating a uniform random vector of Zernike magnitudes up to the 10th radial order (Noll indices: 5-66), we skew the distribution by multiplying each polynomial by c^{n-1}, where n is the Zernike radial order and c is a positive constant ($c < 1$). We found that a c value of 0.85 yields a Zernike coefficient distribution closely aligned with real experimental ground-truth data derived from bead-based calibration measurements [3]. Finally, the L^2 norm of the resulting Zernike vector is normalized to the desired magnitude among mild (0.5 rad), strong (1.0 rad), or severe (1.5 rad). If we consider an engineered PSF, any Zernike magnitudes describing the engineering (in our case, a cylindrical PSF described by an additional value of $Z_6 = 1.0$ rad) are added afterwards.

4. PRE-SELECTION OF SMLM IMAGES FOR BLIND DECONVOLUTION

The effectiveness of the deconvolution algorithm is directly tied to the quality of the images used, making careful selection of the dataset a critical step. To identify an optimal subset, raw SMLM images at different planes are cropped and fitted using an assumed aberration-free PSF. We measured the nanorulers in Fig. 4 with astigmatic imaging, so images were fitted with a PSF with Z_6 = 1 rad defocused by 0.15 $\lambda_{em} + 0.6\, z_{max} \approx 150$ nm [4]. For the NPC experiments in Figs. 5 and 6 of the main document we used defocus imaging. The ideal model is an aberration free PSF defocused by 0.5 $\lambda_{em} + 0.8\, z_{max} \approx 700$ nm [4].

The fitting procedure generates estimates for 3D positions, signal s, and background BG. While using an incorrect PSF model may introduce a quantitative inaccuracies, the resulting distributions should retain the qualitative characteristics of the ground truth, i.e. Gaussian spatial distributions and Poissonian photon statistics. Leveraging this knowledge, we apply filters to exclude molecule images with poor SBR, double emitters (indicated by excessively high signals), or localizations that fail to converge with the assumed ideal model during the fitting procedure.

This pre-selection ensures that only high-quality images are used in the blind deconvolution process, enhancing the robustness and accuracy of the results.

5. FINDING OPTIMAL AUXILIARY PLANES

If the imaged molecules of a sample show only small positional variations along the axial direction compared to the axial PSF stretch, the task of jointly estimating PSF shape and localizations becomes ill-conditioned, and additional image series at different defocus settings should be taken. Here we explain in detail how to identify optimal axial plane settings for this purpose.

The underlying concept of finding ideal imaging planes is to maximize the determinant of the total Fisher information (FI) matrix with respect to the parameters of interest. In our case these

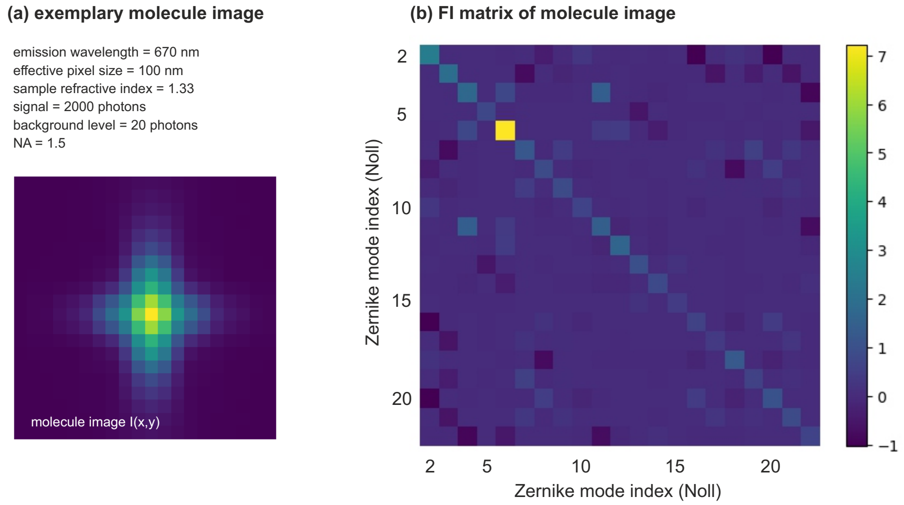

Fig. S1. Fisher information in a single molecule image. (a) Image of a molecule sitting at the glass/buffer interface. The PSF has been made astigmatic to allow for 3D localization. (b) Fisher information matrix of the molecule with regard to all Zernike Noll coefficients ranging from 2 ("tip") to 22 ("secondary spherical aberration").

parameters are the Zernike mode magnitudes a_p with Noll index p up to the fourth radial order without piston. The total FI matrix is the sum of the FI matrices for single molecule images in all imaged planes:

$$\mathrm{FI}_{\mathrm{tot,p,q}} = \sum_{i=1}^{N_z} \mathrm{FI}_{\mathrm{p,q}}(z_i) = \sum_{i=1}^{N_z} \sum_{m,n=1}^{N_m,N_n} \left(\frac{1}{h_{m,n}(z_i)} \frac{\partial h_{m,n}(z_i)}{\partial a_p} \frac{\partial h_{m,n}(z_i)}{\partial a_q} \right), \tag{S2}$$

where $h_{m,n}(z_i)$ represents the detected number of photons expected in the pixel at the index pair (m,n) of the molecule image taken at plane z_i. The coordinates x_i, y_i are assumed to be zero, this means the molecule appears centered in the image. The molecule images $h_{m,n}(z_i)$ are calculated according to the model outlined in section 1.

In the example sketched here we assume to image a "flat" sample, located directly above the coverslip/buffer interface in an inverted TIRF microscope. Relevant imaging parameters are listed in Figure S1. We choose astigmatic imaging using a cylindrical lens pair in the emission path for 3D SMLM, which introduces a controlled amount of Zernike first order astigmatism $a_6 = 1$ rad. An exemplary in-focus image of a molecule located at the glass coverslip is shown in Fig. S1(a) . The matching FI matrix calculated according to Eq. S2 is shown in (b). Noticeable from the matrix is the high value for primary astigmatism (a_6) at the 5th diagonal entry. This is a consequence of using cylindrical lenses to maximize information about the molecule's z-position, which likewise increases the sensitivity of the PSF to Zernike astigmatism a_6. Given the experimental parameters stated in Fig. S1(a), we pre-calculate FI matrices for all possible defocus values ranging from -1000 nm to +1000 nm in steps of 100 nm. Then, for a given number of z-planes N_z (usually 2 or 4), we search for those combinations of z-planes that maximize the determinant of $\mathrm{FI}_{\mathrm{tot,m,n}}$. These planes are the "auxiliary planes". Additionally, we take the main recording plane into account, i.e., the plane which maximizes molecule position information (which is at position z_{main}), leading to the following formulation of the optimization problem:

$$(z_1, ... z_{N_z-1}) = \mathrm{argmax}_{z_1,...z_{N_z-1}} \left(\det \left(\sum_{i=1}^{N_z-1} \mathrm{FI}(z_i) + r \cdot \mathrm{FI}(\mathrm{z}_{\mathrm{main}}) \right) \right). \tag{S3}$$

Here, r is defined as the ratio of the recording lengths of the main and each of the auxiliary image series, which considers the fact that the main recording contains substantially more images (and thus more energy). We assume $r = 1$ for simplicity, but note that a more detailed investigation could also take varying recording times for each plane's image series into account. We identify optimal sets of z-planes using an exhaustive search among all possible combinations. The results for two auxiliary planes are summarized in Table S1. They have been derived assuming that

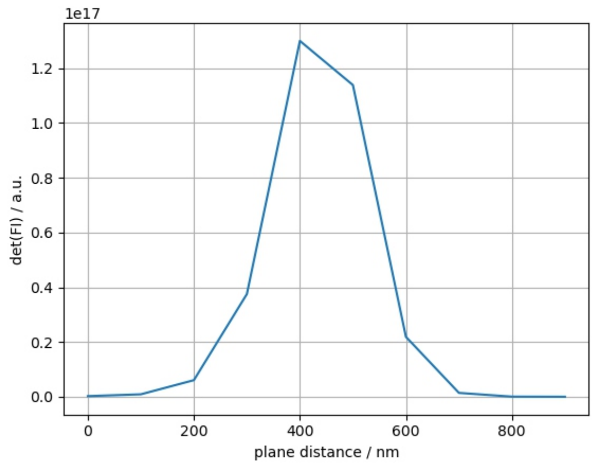

Fig. S2. Determinant of total Fisher information matrix for 3 planes (main plane + 2 auxiliary planes). The Fisher information determinant peaks at at a plane separation between 400 and 500 nm for a very broad range of SBRs.

$z_{\text{main}} = 0$. The position of the most important (primary) auxiliary plane corresponds to the first element in the respective z-tuple, e.g. if the user chooses to take additional data at only one auxiliary plane, the optimal setting for TIRF imaging at a SBR of 10 is $z = 500$ nm. In general we found only a weak dependence of the optimal plane positions on the SBR, as apparent from the table. Expectantly, optimal auxiliary plane positions move more out-of-focus for higher SBRs, where the PSF is more detailed and information-rich when the background level is low. In the TIRF case, we notice that the auxiliary planes lie not exactly symmetric around zero, which is probably due to supercritical angle fluorescence contributions making the PSF asymmetric with respect to the $z = 0$ plane. However, this asymmetry is not very pronounced, and for the sake of simplicity we choose auxiliary planes that lie symmetrically around $z_{main} = 0$, in which case we can easily visualize the total FI matrix determinant for $N_z = 3$ planes in a single plot as shown in Fig. S2. The plot has been calculated for SBR = 100 (s = 5000 photons, BG = 50 photons per pixel) and indicates that an optimal choice of auxiliary planes lies at $z = \pm 400$ nm.
For the case of four auxiliary planes, we again simplified the search by assuming that also the second plane pair is symmetrically arranged around $z_{main} = 0$. The axial positions of these planes are found to be around ± 200 nm for the TIRF case, which is the value we have chosen in the experiment, and ± 400 for the case of imaging with a water immersion lens (NA = 1.0) . However, we note that the exact values of the ideal plane positions depend on many factors assumed in the PSF modeling, such as the assumed SBR or the size to which molecule images are cropped.

Table S1. Optimal positions of auxiliary planes for different SBRs. Values are in nm. Results are shown for TIRF imaging using a high NA oil immersion lens and index-matched imaging in aqueous buffer (RI = 1.33), e.g. using a water-dipping lens.

imaging mode	aux. planes pos. (SBR=10)	aux. planes pos. (SBR=300)
TIRF (NA = 1.5)	500, -300	500, -400
BULK (NA = 1.0)	900, -900	-1200, 1200

Of note, a high Fisher information is not yet a guarantee that the inverse problem can be solved. The FI relates to the curvature of the negative log-likelihood function around its minimum and therefore quantifies the achievable precision of parameter estimates in the presence of noise, but of course the optimization algorithm has to reach the vicinity of this minimum in the first place. It is possible that other, close local minima prevent the algorithm from finding the global minimum, in which case the estimates will be erroneous.

To investigate if imaging at the identified optimal planes results in a true advantage for parameter estimation, we conducted a series of Monte Carlo simulations, in which we estimated

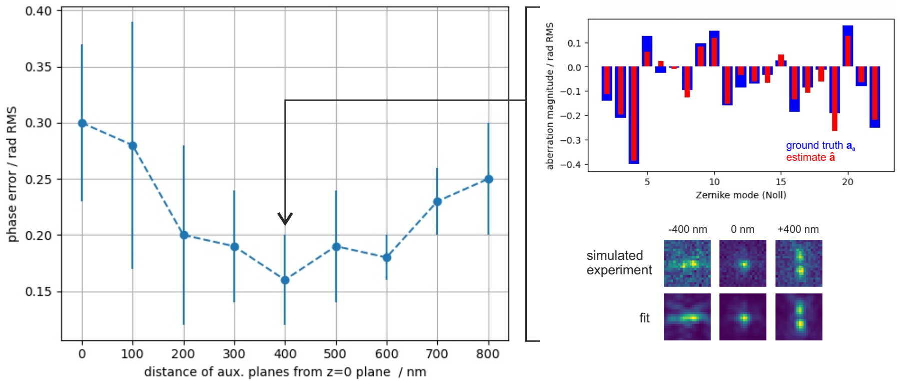

Fig. S3. Finding optimal z-positions for auxiliary planes. The plot shows residual errors after estimating aberrations from a set of 3 images.

a pre-defined aberration mix, described by the vector $\mathbf{a_0}$, from simulated molecule images. For this, a total magnitude of $|\mathbf{a_0}| = 1$ rad was equally randomly distributed over all 21 considered Zernike modes. Different sets of "experimental" images were then calculated, each consisting of three images taken at planes with z-spacings ranging from 0 to 900 nm. For example, the set with z-spacing 0 consists of three images of the same molecule, each taken at the in-focus plane at $z = 0$ nm. The image set with z-spacing 500 nm consists of three molecule images taken at $z = -500$ nm, $0, +500$ nm. Each image was calculated under the assumption of 10k signal photons distributed in the focal plane and a background level of 100 photons (SBR = 100).

The estimation of $\mathbf{a_0}$ is done by an accelerated gradient descent algorithm that minimizes the negative log-likelihood ratio $\text{LLR} = 2\left(L(\hat{\mathbf{a}}) - L(\mathbf{a_0})\right)$, where $\hat{\mathbf{a}}$ is the current estimate for the vector of Zernike mode magnitudes. $L(\mathbf{a})$ is the log-likelihood for the Zernike vector $\mathbf{a}$:

$$L(\mathbf{a}) = \sum_{i=1}^{N_z=3} \sum_{m,n=1}^{N_m,N_n} \left(I_{m,n}(z_i, \mathbf{a}) - I_{m,n}(z_i, \mathbf{a_0}) \log\left(I_{m,n}(z_i, \mathbf{a})\right)\right) \tag{S4}$$

The results of the simulated experiment are summarized in Fig. S3. They confirm that recording data at additional auxiliary planes leads to smaller errors in the estimated Zernike coefficients, and that a z-spacing of 400 to 500 nm is about optimal. Each data point in the plot represents the mean residual RMS error in the objective pupil phase function $\Phi(\mathbf{r})$, where the aberrations have been modeled:

$$\epsilon = \sqrt{\frac{1}{A} \sum_i \left(\Phi_{\hat{\mathbf{a}}}(\mathbf{r_i}) - \Phi_{\mathbf{a_0}}(\mathbf{r_i})\right)^2}, \tag{S5}$$

where A is the area of the objective pupil. The error bars show the standard deviation of a measurement, which we derived from five independent trials assuming different aberration mixes.

REFERENCES

1. D. Axelrod, "Fluorescence excitation and imaging of single molecules near dielectric-coated and bare surfaces: a theoretical study," J. microscopy **247**, 147–160 (2012).
2. H. Ortkrass, M. Müller, A. K. Engdahl, *et al.*, "High sensitivity cameras can lower spatial resolution in high-resolution optical microscopy," Nat. Commun. **15**, 8886 (2024).
3. M. C. Schneider, F. Hinterer, A. Jesacher, and G. J. Schütz, "Interactive simulation and visualization of point spread functions in single molecule imaging," Opt. Commun. **560**, 130463 (2024).
4. P. Zelger, L. Bodner, M. Offterdinger, *et al.*, "Three-dimensional single molecule localization close to the coverslip: a comparison of methods exploiting supercritical angle fluorescence," Biomed. Opt. Express **12**, 802–822 (2021).
